# High‐Density Dynamic Culture of Neonatal Porcine Islets Improves Islet Recovery and Function Post‐Transplantation

**DOI:** 10.1111/xen.70181

**Published:** 2026-11-11

**Authors:** Summer A. Helmi, Chelsea Castro, Soren Stachniak, Arghyadip Bose, Abby Basiuk, Mandy Rosko, Andrew R. Pepper, Gregory S. Korbutt

**Affiliations:** ^1^ Department of Surgery University of Alberta Edmonton Alberta Canada; ^2^ Alberta Diabetes Institute Edmonton Alberta Canada

**Keywords:** Islet engraftment, neonatal porcine islets, scale‐up manufacturing, Xenotransplantation, β cell replacement therapy

## Abstract

**Introduction:**

Type 1 diabetes (T1D) results from autoimmune destruction of pancreatic β‐cells, necessitating lifelong exogenous insulin therapy. Scalable β‐cell replacement strategies are needed to reproducibly increase ß cell production. Neonatal porcine islets (NPIs) are a promising xenogeneic source of ß cells; however, limited manufacturing scalability hinders clinical translation. This study optimizes a dynamic culture system to assess its potential for scalable NPIs production for clinical translation.

**Methods:**

NPIs were cultured under three conditions: static suspension and dynamic suspension in a PBS Mini Vertical‐Wheel bioreactor at 4.5K (Bio 4.5) and 6.0K (Bio 6.0) NPIs/ml. NPI recovery and composition were evaluated via total DNA, insulin content, and β‐cell recovery. Functional and structural assessments included glucose‐stimulated insulin secretion (GSIS), mitochondrial oxygen consumption rate (OCR), and immunohistochemistry. Streptozotocin‐induced diabetic B6.129S7‐*Rag1^tm1Mom^*/J mice received transplants of NPIs from static or Bio 6.0 cultures. Metabolic function was assessed by non‐fasting glucose and IPGTT, alongside histological analysis of explanted grafts.

**Results:**

Bio 6.0 NPIs demonstrated significantly higher DNA (*p* < 0.001), insulin content *(p* < 0.0001), and β cell recovery *(p* < 0.01), with increased β‐cells per pancreas (*p* < 0.05), smaller aggregate size (*p* < 0.0001), and improved morphology. In vivo, Bio 6.0 grafts achieved faster glycemic normalization (*p* < 0.05) and improved IPGTT outcomes at 10 weeks (*p* < 0.05). Histology confirmed cohesive architecture and strong insulin staining.

**Conclusion:**

High‐density bioreactor culture enhances NPIs performance and the potential for scalability, improving diabetes reversal and supporting clinical translation for β cell replacement therapy.

## 1 Introduction

Type 1 diabetes (T1D) is characterized by the selective immunogenic destruction of pancreatic β cells leading to hyperglycemia [1, 2, 3]. This drastic immune reaction leaves people living with diabetes dependent on exogenous insulin for survival [4]. Currently, T1D affects over 9 million people worldwide, with an annual increase of 3%–4% [5]. Advances in exogenous insulin delivery still fall short of replicating the physiological regulation of blood glucose levels [6]. This leaves the patient susceptible to life‐threatening complications and chronic systemic conditions, including cardiovascular disease, retinopathy, nephropathy, and more [7, 8, 9]. These limitations call for a treatment that can emulate the physiological process of insulin production.

Cell replacement therapies offer an ideal solution for T1D by replacing damaged β cells and creating a balanced microenvironment that supports the function and survival of transplanted islets. Research has introduced various cell sources, delivery techniques, and transplantation sites [10, 11, 12, 13, 14]. Whole pancreas transplantation has successfully achieved normoglycemia in recipients; however, limited donor availability, combined with surgical risks and the need for lifelong immunosuppression, hinders the broader use of this approach [15]. The groundbreaking Edmonton Protocol, introduced in 2000, involved injecting isolated human islets into the portal vein, demonstrating insulin independence with a lifelong immunosuppression regimen [16, 17]. Although this protocol marked a significant advancement in treating T1D, it remains constrained by the low number of human donors, as it requires at least two pancreata per recipient, along with challenges related to immune rejection and post‐transplantation islet survival [18, 19, 20].

Recent progress in regenerative medicine has enabled stem cell‐derived islets to replace damaged primary islets. Induced pluripotent stem cells (iPSCs) have shown promise in generating functional β‐like cells capable of glucose‐responsive insulin secretion [21, 22, 23]. Reports of insulin independence following the transplantation of islets derived from stem cells in patients encourage further research to address the significant challenges associated with this therapy [11, 13, 24, 25]. These challenges include off‐target differentiation effects, high production costs, and immune rejection, which have heightened interest in xenotransplantation as a viable alternative [26, 27, 28].

Porcine pancreatic islets have been proposed as a potential replacement therapy due to their physiological compatibility with human metabolism [29, 30, 31, 32]. Moreover, genetically modified pigs can significantly reduce immunogenicity [33]. Neonatal Porcine Islets (NPIs) are easier and less expensive to isolate than adult pig islets, addressing the availability issue by providing a limitless cell source and lower costs for animal housing and *ex vivo* cell processing compared to human and induced pluripotent stem cell islets [34, 35, 36, 37, 38]. This makes them ideal candidates for clinical translation. However, several barriers still prevent NPIs from reaching clinical application. A key barrier is scalability; current static culture systems are unsuitable for large‐scale production of functional NPIs due to apoptosis, dedifferentiation, and reduced viability [39, 40, 41, 42]. Additionally, current static culture systems are labor‐intensive and susceptible to human error [37, 43]. Dynamic cell culture systems have been proposed for 3D cell cultures to overcome these challenges [44, 45]. Vertical wheel bioreactors have been effectively utilized for the scalable production of islet‐like aggregates from human iPSCs. This bioreactor system has eliminated the need for traditional two‐dimensional culture steps across all stages of iPSCs expansion, islet differentiation, and maturation. This approach has produced large, uniform, and viable islet‐like aggregates with consistent size and cell composition while maintaining high cell viability [24, 46, 47, 48, 49]. By adopting this dynamic cell‐culture approach, this study examines its potential to culture NPIs at high density for subsequent future scalable NPIs manufacturing, supporting the production of larger quantities of NPIs.

## 2 Materials and Methods

### 2.1 Preparation of Neonatal Porcine Islets

This study was conducted in accordance with the guidelines of the institutional ethical committee of the University of Alberta and the Canadian Council on Animal Care. NPIs were obtained from 1‐ to 3‐day‐old male and female Duroc neonatal piglets (University of Alberta Swine Research Center, Edmonton, AB, Canada) [32, 50, 51]. NPIs were isolated and cultured overnight in Ham F10 media (Sigma‐Aldrich) with 666 µL/L protease inhibitor P8340 (Sigma‐Aldrich) and 5 µg /mL FMK001 caspase inhibitor (R&D Systems). The next day, NPIs were then resuspended in Ham's F10 media with protease inhibitor and 10 µM ROCK inhibitor Y27632 (Sigma‐Aldrich) and incubated for 2 days. On day 3 of culture post‐isolation, NPIs intended for differentiation were washed in calcium‐ and magnesium‐free Hank's Balanced Salt Solution (HBSS) [32, 50].

On day 3 post‐isolation, following washing with calcium and magnesium‐free Hank's Balanced Salt Solution (HBSS), NPIs were manually counted and split into two primary culture systems for further enrichment and analysis. 1‐ Conventional Static Suspension Culture: NPIs were seeded into low‐attachment cell culture dishes at a density of 2000 islet aggregates per milliliter in a total volume of 30 mL of media per dish, as per our standard protocol [32]. 2‐ Vertical Wheel Bioreactor Culture: Two parallel PBS mini vertical wheel bioreactors were utilized, with NPIs seeded at densities of 4500 (Bio 4.5) and 6000 (Bio 6.0) NPIs per ml, respectively. Both bioreactors were operated at a rotation speed of 40 RPM to maintain aggregate suspension and promote uniform nutrient distribution (Figure 1A). Media changes were performed according to the stepwise enrichment protocol previously established (Figure 1B) [50, 51, 52]. NPIs from the three culture conditions were collected at the end of the enrichment period for characterization/ transplantation.

**FIGURE 1:**
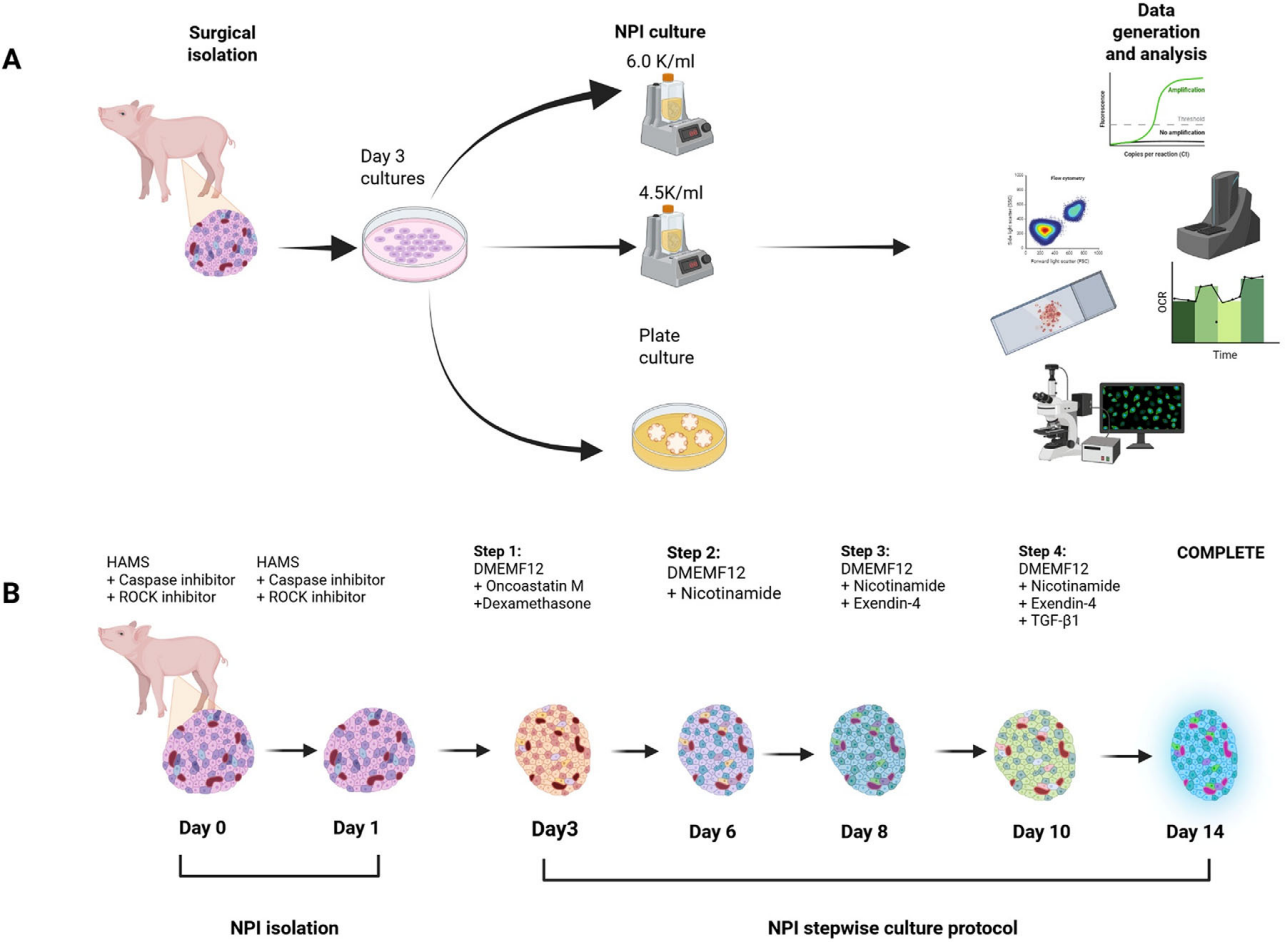
Experimental workflow and enrichment protocol for neonatal porcine islet (NPI) culture and analysis. (A) Schematic overview of the experimental methodology. (B) Timeline of the NPI enrichment protocol showing media composition and growth factors at each stage.

### 2.2 Characterization of Neonatal Porcine Islets

NPIs harvested from each experimental group were fixed in 10% neutral‐buffered formalin, embedded in agarose to maintain aggregate integrity, and subsequently processed for paraffin embedding. Blocks were sectioned at a thickness of 5 µm for immunohistochemical analysis. Immunostaining was performed according to established protocols, with primary antibodies targeting insulin (Dako, A0564), cytokeratin 7 (CK7) (Dako, M7018), and glucagon (Sigma‐Aldrich, G2654).

For detection, species‐compatible secondary antibodies were used at the dilution of 1:200, either biotinylated for chromogenic detection or Alexa Fluor‐conjugated (Invitrogen, A11073, Invitrogen, A11076) for immunofluorescence. The biotinylated secondary antibodies were visualized using the Avidin‐Biotin Complex (ABC) method with 3,3’‐diaminobenzidine (DAB) as the chromogen (Biolegend 926506), followed by counterstaining with hematoxylin and eosin (H&E). For immunofluorescence, the slides were mounted with Prolong Gold Antifade reagent containing DAPI (Invitrogen, P36931) for nuclear visualization. Newborn pig pancreas sections served as positive controls, while negative controls included sections incubated with secondary antibodies in the absence of primary antibody [32, 50, 52].

Cellular DNA content was measured using the CyQUANT Cell Proliferation Assay Kit (Invitrogen, C7026), following the manufacturer's protocol. Briefly, NPIs aggregates were lysed with cell lysis buffer (Thermo Fisher Scientific, EN0531), and the lysates were incubated with CyQUANT GR dye for three hours. Fluorescence intensity was measured using a microplate reader. DNA concentration in each sample was determined by comparison to a standard curve generated from serial dilutions of bacteriophage λ DNA. Insulin content was measured via electrochemiluminescence (MesoScale Discovery) according to the manufacturer's instructions [32]. DNA and insulin recovery rates were quantified and expressed as percentages relative to the initial input. Fold changes were calculated for each culture condition (static plate, 4500 aggregates/mL bioreactor, and 6000 aggregates/mL bioreactor). NPIs from each condition were dissociated using TrypLE Select dissociation solution (Gibco, A12177‐01), and the single cells were centrifuged at 500 g for 5 min. The cell pellets were resuspended in 1000 µL of the Fixation and Permeabilization Solution (Z fix solution; BD Biosciences) and then incubated for 20 min at room temperature. After that, the cells were washed with 10 mL of wash buffer and centrifuged at 700 × g for 5 min. Staining was performed using anti‐insulin FITC (Novus Bio, NBP2‐34780F), anti‐cytokeratin 7 (Abcam, ab210628), and mouse anti‐glucagon (Sigma‐ Aldrich, G2654) antibodies for 1 h at 4°C in the dark, and isotype controls were included for each marker. Analysis was performed using the BD FACS Canto II flow cytometer (BD Biosciences), with appropriate compensation and gating to quantify β cells, α cells, and CK7‐positive cells as percentages of the total cell population [50].

The glucose‐stimulated insulin secretion (GSIS) assay was performed using sequential glucose stimulation. Fresh solutions were prepared for each experiment as follows: glucose‐free RPMI 1640 base medium (G0) ((Invitrogen, #11879‐020) supplemented with 0.5% bovine serum albumin (BSA) (Sigma Aldrich, A7906), penicillin/streptomycin (Gibco, 15070063), and HEPES buffer was used as the foundation for 5.6 mM (overnight incubation), 2.8 mM (low glucose), 20.0 mM (high glucose). Islets were incubated overnight with 5.6 mM glucose solution. The next day, the islets were sequentially stimulated for 1 h per solution, and the supernatants were collected after each stimulation (2.8 mM, 20 mM) for insulin quantification [32]. The stimulation index (SI) was calculated as the ratio of insulin secreted during high‐glucose stimulation to low‐glucose stimulation. Insulin values were normalized to total insulin content, and the data were presented as a percentage.

The metabolic activity of NPIs was assessed using the Seahorse XFe24 Extracellular Flux Analyzer (Aglient).  70 NPIs/well were hand‐picked and loaded into the islet capture microplate. The picked islets were incubated in MA media (DMEM supplemented with 1% FBS, 2.8 mM D‐glucose, 2 mM sodium pyruvate, and 2 mM L‐glutamine) for one hour. The assay was performed with 16 mM glucose, 5 µM oligomycin, 3 µM FCCP, and 5 µM rotenone/antimycin. Oxygen consumption rate (OCR) and extracellular acidification rate (ECAR) were measured in real time at multiple intervals using the Seahorse Wave software, and data were normalized to islet count /well [53, 54].

### 2.3 Neonatal Porcine Islet Gene Expression

To assess NPIs gene expression, total RNA was isolated from NPIs using TRIzol reagent (Invitrogen, Thermo Fisher Scientific, 15596026) according to the manufacturer's instructions. RNA was purified using RNeasy Mini Kit (Qiagen, 74116) as follows: NPIs were lysed in TRIzol, incubated at room temperature for 20 min, and then stored at −80°C until extraction. For RNA extraction, samples were thawed, mixed with chloroform, and then centrifuged at 12 000 rpm for 15 min at 4°C to separate the aqueous phase. Next, RNA‐containing upper phase was transferred to new tubes, mixed with 75% ethanol, and loaded onto RNeasy spin columns. Then, the columns were washed sequentially with buffers, and the RNA was eluted in RNase‐free water. RNA concentration and purity were assessed using a NanoDrop spectrophotometer (Thermo Fisher Scientific). For cDNA synthesis, 1000 ng of total RNA was reverse transcribed using the Applied Biosystems cDNA Archive Kit (Thermo Fisher Scientific‐K2562) in a 10 µL reaction volume, according to the manufacturer's instructions. Quantitative real‐time PCR (qRT‐PCR) was performed using TaqMan Fast Advanced Master Mix (Applied Biosystems, Thermo Fisher Scientific,4444558) and validated gene‐specific TaqMan probes (Table 1). The qPCR reactions were run on a StepOne Plus real‐time thermocycler (Applied Biosystems, Thermo Fisher Scientific). Relative gene expression quantification (RQ) was calculated and normalized to GABDH as a housekeeping gene and undifferentiated NPIs as a reference sample [50, 52].

**TABLE 1:** Validated porcine taqman primer and probe sets.

Gene	Gene symbol	Catalog number
Pancreatic duodenal homeobox 1	*PDX1*	Ss03373351_m1
Insulin	*INS*	Ss03386682_u1
Glucagon	*GLUC*	Ss03384069_u1
Paired box 6	*PAX6*	Ss03397583_m1
NK6 homeobox 1	*NKX6‐1*	Ss03373352_m1
SRY‐box9	*SOX9*	Ss03392406_m1
Pancreatic polypeptide	*PPY*	Ss03375477_u1
Somatostatin	*SST*	Ss03391856_m1
Glyceraldehyde‐3‐phosphate dehydrogenase	*GAPDH*	Ss03375629_u1

### 2.4 Transplantation and Metabolic Follow‐up

Male and female B6.129S7‐*Rag1^1Mom^*/J mice (Jackson Laboratories, strain # 002216) were used as recipients of NPIs under the kidney capsule transplants at 3000 NPIs/transplant. All mice were maintained under pathogen‐free conditions in a climatized room with free access to water and pelleted food. Diabetes was induced in the mice through intraperitoneal injections of 185 mg/kg streptozotocin freshly dissolved in acetate buffer 2 weeks before transplantation. Male and female mice were randomly distributed between experimental groups [55]. Blood samples were obtained from the tail vein for glucose measurement (OneTouch UltraMini glucose meter. The static suspension culture condition (*n* = 6) or Bio 6. condition NPIs (*n* = 18) were transplanted under the left kidney capsule as previously described [32, 56, 57]. The static suspension culture condition served as the benchmark control, and based on NPIs post‐culture recovery, only NPIs cultured in the Bio 6.0 condition were used in transplant studies. NPIs were aspirated into polyethylene tubing (PE‐90) and pelleted by centrifugation, then gently placed under the kidney capsule with the aid of a micromanipulator syringe (Figure 2).

**FIGURE 2:**
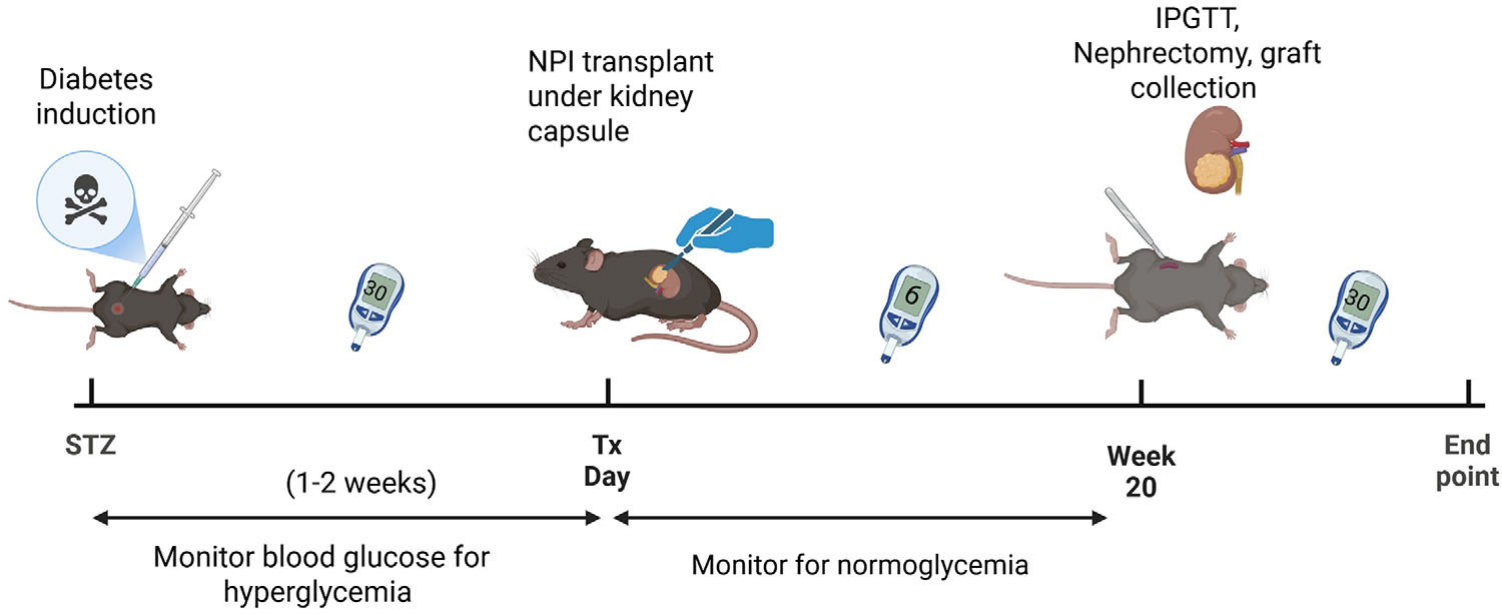
Schematic diagram illustrating the methodology for isolating and transplanting Neonatal Porcine Islets.

All mice were monitored for non‐fasting blood glucose levels once per week. When the blood glucose level was 11.0 mmol/L or less for 2 consecutive weeks, mice were considered normalized. An intraperitoneal glucose tolerance test (IPGTT) was performed at 10 weeks and at 20 weeks post‐transplantation. After a 12‐hour fast, D‐glucose (3 g /kg body weight) was administered intraperitoneally into non‐anesthetized mice. Blood glucose was monitored at 0, 15, 30, 60, 90, and 120 min, and blood samples were obtained from the tail vein at 0 and 60 min. Serum porcine insulin concentrations were measured at fasting (T0) and 60 min after intraperitoneal glucose injection (T60). One week post 20‐week IPGTT, all mice underwent a survival nephrectomy of the graft‐bearing kidney, which was taken for morphological and histological assessments and graft insulin content extraction. Nephrectomized animals were subsequently monitored twice to confirm the return of hyperglycemia before they were humanely euthanized [32, 57].

### 2.5 Statistical Analysis

Static suspension and Vertical‐Wheel bioreactor cultures were conducted using NPIs from 5 independent isolations (*N* = 5). Each independent isolation was obtained from a total of 8 male or female neonatal pancreata. On day 3 post‐isolation, NPIs from all 8 pancreata were pooled and then allocated into either the static and bioreactor culture conditions (total of 3 conditions). At the end of the enrichment period (day 14, Figure 1B), total DNA and insulin contents, GSIS, and OCR assays were assessed from triplicate aliquots of the islet cell suspensions (*n* = 3). Subsequently, values from the triplicate aliquots were averaged for statistical analysis. Gene‐expression analysis was performed in 4 of the independent isolations (*N* = 4). To assess aggregate size, we analyzed 11 bright‐field images per condition distributed across the 5 independent isolations (*n* = 11).

Data are expressed as mean ± SEM. Statistical significance was calculated using 1‐way ANOVA followed by Tukey's multiple‐comparisons test or a two‐tailed unpaired Student's t‐test. Graft survival was defined as the proportion of mice maintaining normoglycemia (blood glucose < 11.0 mmol/L for two consecutive weeks). Survival curves were constructed using the Kaplan‐Meier method and compared between groups using the log‐rank (Mantel‐Cox) test. Statistical Analysis of weekly non‐fasting blood‐glucose values were analyzed using two‐way repeated‐measures ANOVA, with treatment group and time as factors, followed by Šídák's multiple‐comparisons test. *p‐*values <0.05 was considered statistically significant. Statistical analyses were performed using GraphPad Prism software.

## 3 Results

### 3.1 Effect of Neonatal Porcine Islet Culture in the PBS Mini Vertical‐Wheel Bioreactor

Culture of NPIs in the PBS Mini Vertical‐Wheel Bioreactor at a seeding density of 6000 IEQ/mL (Bio 6.0) resulted in enhanced DNA and insulin recovery compared to both control culture and bioreactor culture at 4500 IEQ/mL (Bio 4.5). DNA and insulin contents increased significantly in Bio 6.0 compared to control (*p* < 0.001). Fold changes in DNA and insulin were also highest in Bio 6.0 (*p* < 0.05, *p* < 0.01), respectively (Table 2).

**TABLE 2:** **Comparison of DNA and insulin recovery rates across different culture conditions**.

	% Content loaded on day 3		Fold change to control	
Condition	DNA	Insulin	DNA	Insulin
**Control**	17.45 ±2.14	100 ± 14.85	1	1
**Bio 4.5**	20.42 ±2.76	133.30 ± 29.29	1.42 ± 0.29	1.18 ± 0.20
**Bio 6**	34.14± 1.31^***^	244.14 ± 20.44^***^	2.21 ± 0.31^*^	2.31 ± 0.23^**^

Values are expressed as mean ± SEM of *N* = 5, *n* = 3

* = *p* < 0.05.

** = *p* < 0.01.

*** = *p* < 0.001 vs. control.

Flow cytometry analysis and immunofluorescence staining/imaging demonstrated that β cells, α cells, and CK7‐positive ductal cells proportions remained consistent in control as well as bioreactor groups. β cells constituted approximately 22%–23% of the total cell population, α cells ranged from 36%–44%, and CK7‐positive ductal cells were below 5% in all groups (Table 3).

**TABLE 3:** Comparison of neonatal porcine islets’ cellular composition following differentiation.

Cell composition (% total)				% cell recovery / total cells loaded on day 3			β cells/ Pancreas X 10^6^
Condition	β cells	α cells	CK7 cells	β cells	α cells	CK7 cells	
**Control**	22.88 ± 2.30	36.71 ± 3.42	3.77 ± 1.36	80.82 ± 4.72	46.63 ± 9.97	2.77 ± 0.88	4.18 ± 0.96
**Bio 4.5**	22.11± 3.08	44.41 ± 5.57	4.40 ± 1.20	87.64 ± 5.50	59.75 ± 10.01	2.73 ± 0.71	4.09 ± 0.76
**Bio 6.0**	22.56 ± 3.90	38.79 ± 8.06	4.99 ± 1.78	129.86 ± 8.692^**^	62.88 ± 1.99	5.23 ± 1.78	8.26^*^ ± 1.12

Values are expressed as mean ± SEM of *N* = 5, *n* = 3,

* = *p* < 0.05,

** = *p* < 0.01 vs. control

Cell recovery, expressed as percentages relative to the initial cell input, revealed that the PBS Mini Vertical‐Wheel Bioreactor, with a 6.0 k/ml seeding density, significantly enhanced β cell recovery vs. control (*p* < 0.01), accompanied by modest increases in α cells and CK7‐positive cells (Table 3). Moreover, the Bio 6.0 group showed significantly higher β cell/ pancreas compared to control (*p* <0.05) (Table 3).

Examining NPIs morphology through bright‐field and confocal imaging revealed a significant decrease in islet aggregate size in the Bioreactor groups compared to the control (*p* <0.0001) (Figure 3A). Immunofluorescence confirmed the presence of insulin and glucagon‐positive cells within the aggregates, with low expression of CK7‐positive cells. Additionally, chromogenic immunohistochemistry for insulin using DAB showed comparable insulin expression across conditions (Figure 3B).

**FIGURE 3:**
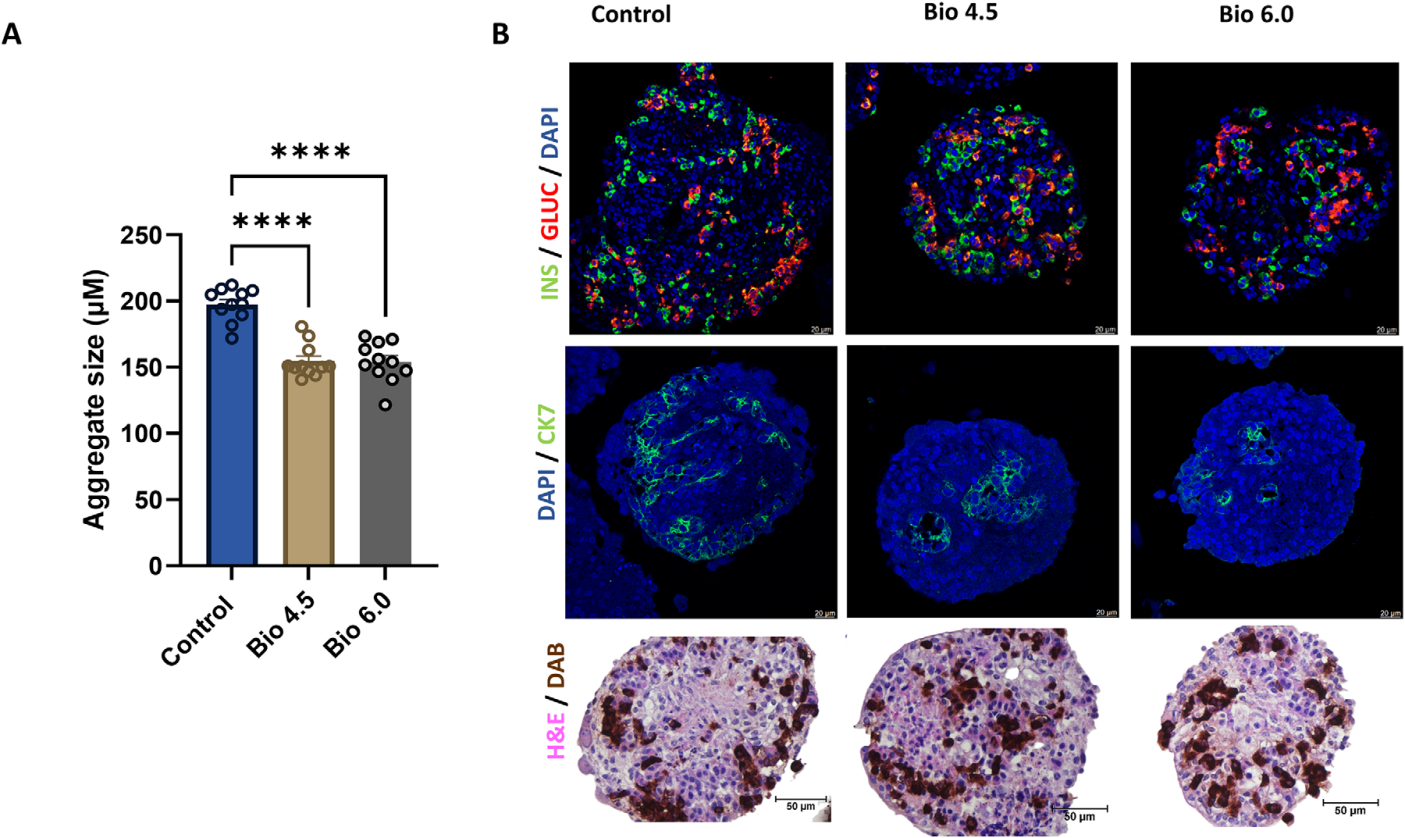
Histology and Immunohistochemistry Staining of Neonatal Porcine Islets. (A) Quantitative comparison of NPIs size across culture conditions (control, Bio 4.5, and Bio 6.0; *n* = 11, **** = *p* < 0.000. (B) Representative immunofluorescence images showing co‐expression of insulin (green) and glucagon (red) in NPIs (40x), scale bar 20 µm. Immunofluorescence staining for CK7 (green) in NPIs (40x), scale bar 20 µm, And Chromogenic immunohistochemistry for insulin using DAB (brown) in NPIs (40 x), scale bar 50 µm.

Glucose‐stimulated insulin secretion (GSIS) assays demonstrated that all groups retained glucose responsiveness, as reflected by the stimulation index (SI). (Table 4).

**TABLE 4:** Glucose‐stimulated insulin secretion of neonatal porcine islets.

% Insulin content			
Condition	2.8 mM glucose	20 mM glucose	Stimulation index
**Control**	0.30 ± 0.15	0.59 ± 0.21	1.96± 0.18
**Bio 4.5**	0.20 ± 0.13	0.31 ± 0.24	1.60 ± 0.05
**Bio 6.0**	0.32 ± 0.15	0.56 ±0.22	1.80 ± 0.09

Values are expressed as mean ± SEM of *N* = 5, *n* = 3

Metabolic profiling revealed that NPIs cultured in the bioreactor at 6k/ml seeding density (Bio 6.0) maintained robust mitochondrial function, represented by a significant increase in the OCR/ECAR ratio (*p* < 0.05) (Figure 4C). There were no differences in the mitochondrial stress test parameters represented by the basal respiration, ATP‐linked respiration, proton leak, or spare respiratory capacity among the groups when normalized to islet number (Figure 4E). Bioenergetic health index (BHI) and glucose‐related bioenergetic (GBHI) health index were similar across conditions as well (Figure 4D). The area under the curve (AUC) for both oxygen consumption rate (OCR) and extracellular acidification rate (ECAR) was similar across all conditions (Figure 4A,B).

**FIGURE 4:**
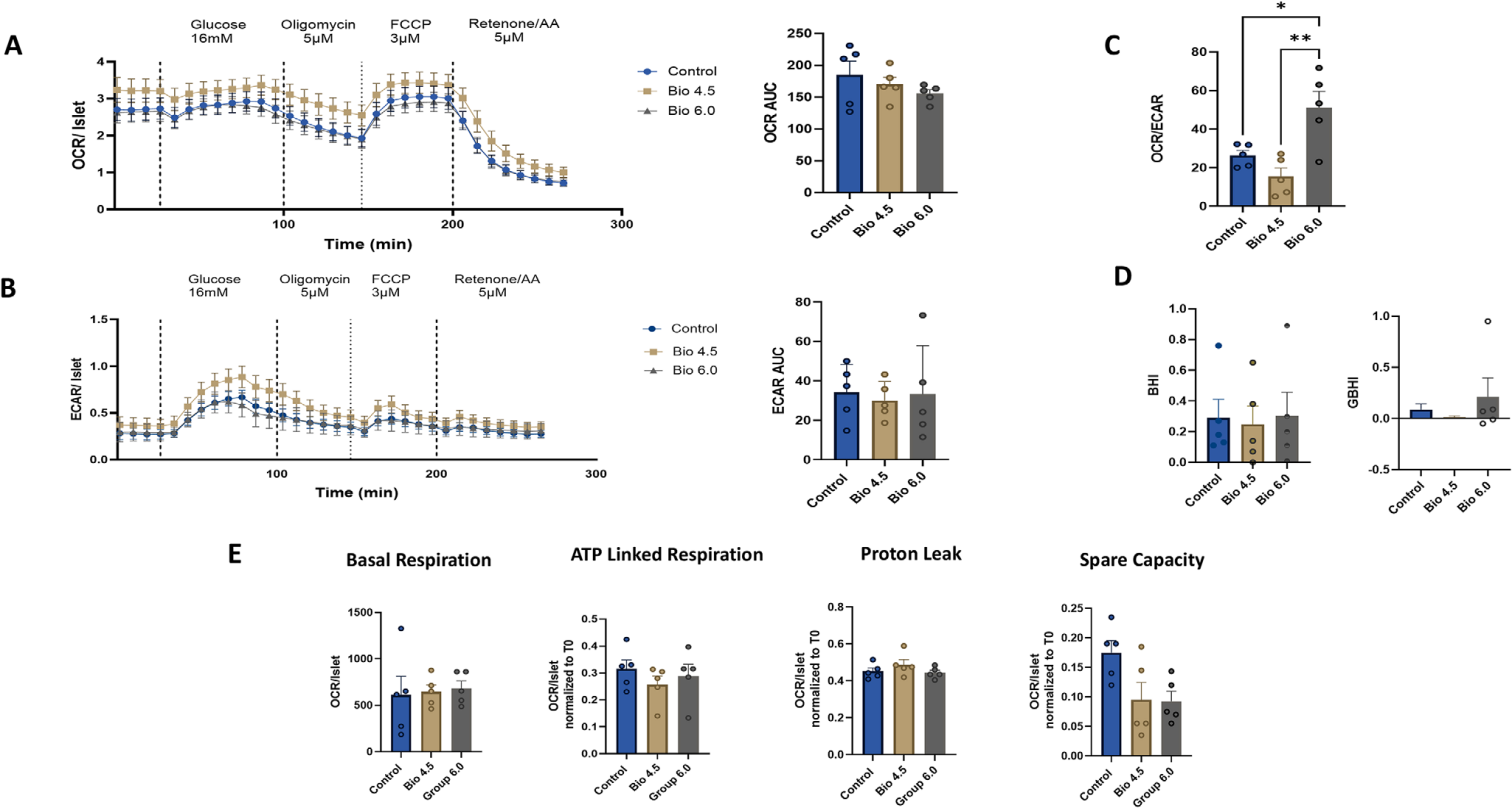
Metabolic Assessment of NPIs. (A) Oxygen consumption rate (OCR) and area under the curve; (B) extracellular acidification rate (ECAR) and area under the curve in NPIs across conditions. (C) OCR/ECAR ratio; (D) Bioenergetic health index (BHI) and glucose‐related bioenergetic health index (GBHI) calculated from mitochondrial stress test parameters. (E) Mitochondrial stress test parameters, including basal respiration, ATP‐linked respiration, proton leak, and spare respiratory capacity, compared between groups.​ *N* = 5, *n* = 3; * = *p* < 0.05, ** = *p* < 0.01.

Quantitative PCR analysis indicated distinct gene expression patterns across culture conditions. Compared with static controls, Bio 6.0 NPIs exhibited significantly increased expression of insulin (INS, p<0.05), glucagon (GCG, p<0.01), somatostatin (SST, p<0.01), and pancreatic and duodenal homeobox 1 (PDX1,p<0.01). In contrast, SOX9 and PAX6 expression were significantly reduced in both the bioreactor groups compared with static controls (p<0.01). NKX6.1 expression was significantly reduced in the Bio 4.5 group compared with controls (p<0.01), whereas Bio 6.0 showed a comparable level to control. Pancreatic polypeptide (PPY) expression was significantly increased in Bio 4.5 compared with controls (p<0.01), while its expression in Bio 6.0 did not differ significantly from controls (Figure 5).

**FIGURE 5:**
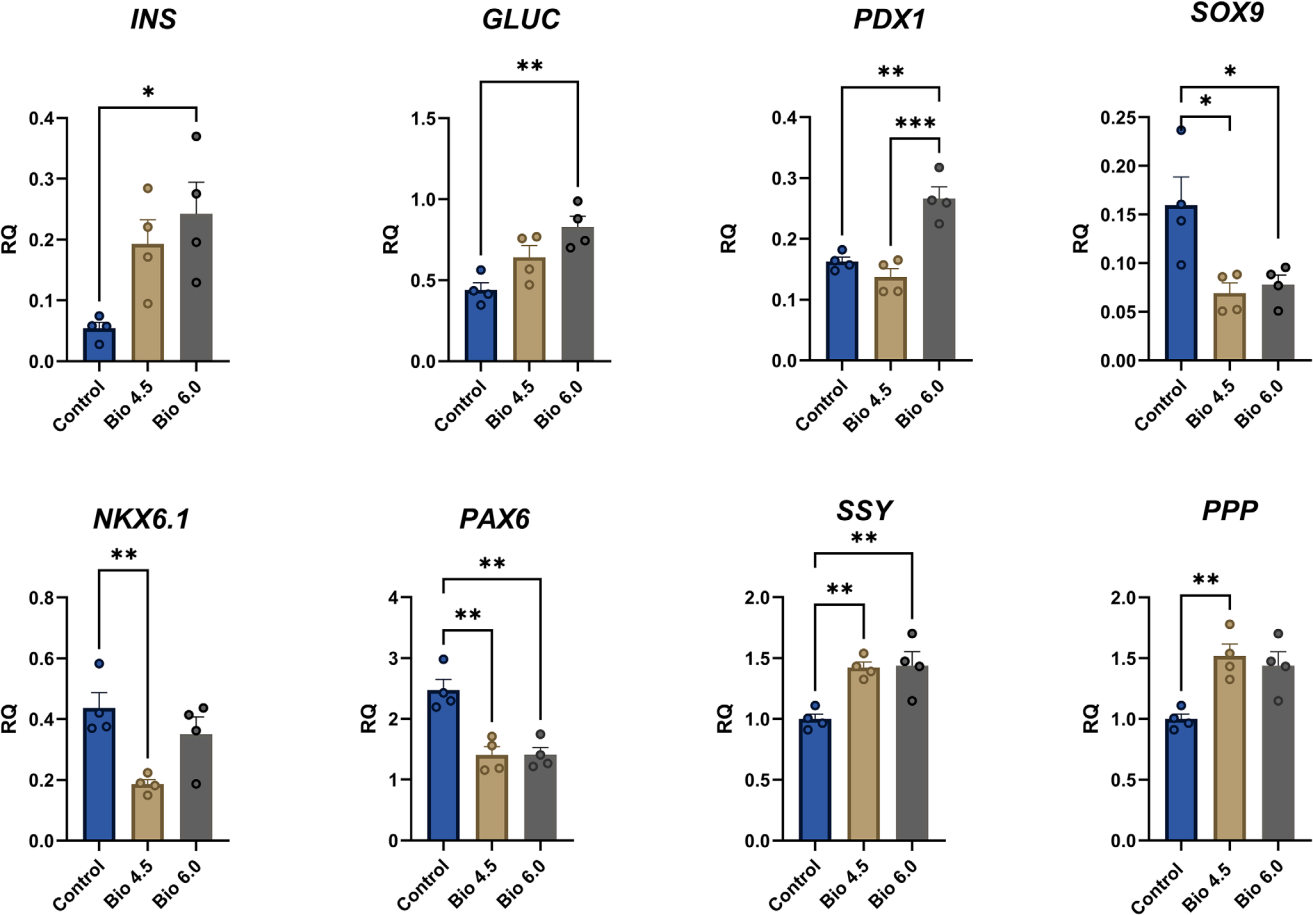
Quantitative PCR Analysis of NPIs Gene Expression. Relative quantification (RQ) of gene expression for insulin (INS), glucagon (GCG), NKX6.1, PAX6, PDX1, SOX9, somatostatin (SST), and pancreatic polypeptide (PPY) in NPIs cultured under control, Bio 4.5, and Bio 6.0 conditions, *N* = 4, *n* = 3; * = *p* < 0.05, ** = *p* < 0.01.

### 3.2 Metabolic Follow‐up of Transplant Recipients

Recipients of Bio 6.0 NPIs demonstrated a progressive and significant decline in blood glucose levels over the 20‐week transplantation period when compared to the control recipients (Figure 6A); these results are reflected by the significantly lower area under the curve (Figure 6B). The proportion of mice achieving normoglycemia (blood glucose < 11.0 mmol/L for two consecutive weeks) was significantly higher in the Bio 6.0 group compared to controls (*p* <0.05) (Figure 6C). In addition, recipients of Bio 6.0‐differentiated NPIs reached normoglycemia significantly faster than control recipients, with shorter mean time to euglycemia (*p* < 0.05) (Figure 6D).

**FIGURE 6:**
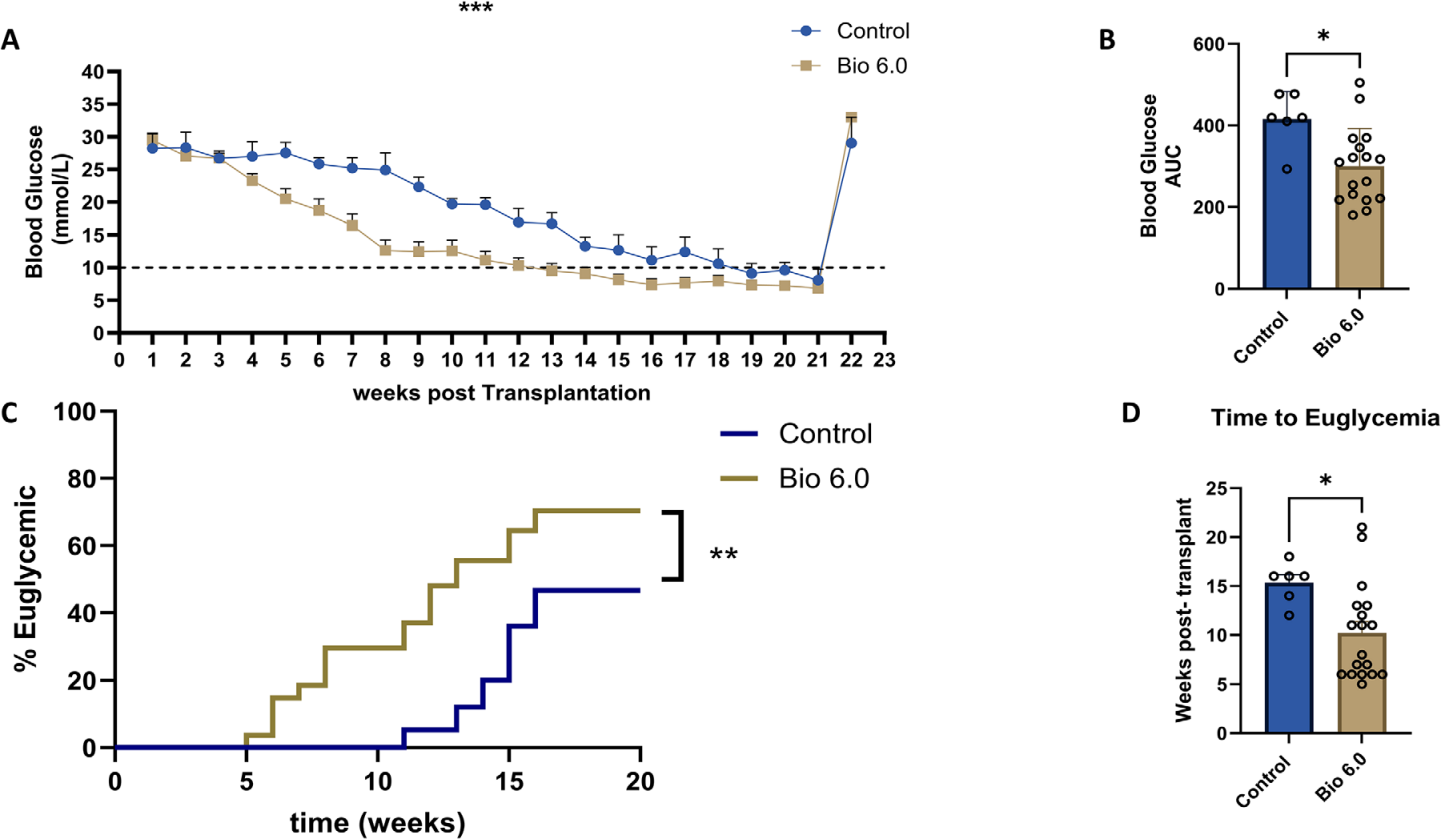
Islet transplantation and metabolic follow‐up of diabetic B6.129S7‐Rag1^tm1Mom^/J–immunodeficient mice transplanted with neonatal porcine islets (3000 NPIs) under the kidney capsule (KC) from Bio 6.0 (*n* = 18) or control (*n* = 6) recipients. (A) Mice were monitored weekly for non‐fasting blood glucose. (B) Glycemic control was quantified as the area under the curve (AUC) of the blood glucose profiles shown in (A). (C) Proportion of mice achieving normoglycemia, defined as blood glucose < 11.0 mmol/L for two consecutive weeks. (D) Time to normoglycemia (weeks post‐transplant) in Bio 6.0 and control groups; * = *p* < 0.05, ** = *p* < 0.01.

An IPGTT was performed at 10 weeks post‐transplantation in all transplanted mice to evaluate glucose homeostasis at an intermediate time point. Recipients of Bio 6.0 NPIs exhibited lower blood glucose readings throughout the IPGTT compared to control recipients, with a significantly lower AUC (*p* < 0.001) (Figure 7A,B). Serum porcine insulin measurements taken at fasting (T0) and 60 min after glucose challenge (T60) revealed that Bio 6.0 recipients had significantly higher circulating NPIs‐derived insulin levels at T60 compared to control recipients (*p* < 0.01) (Figure 7C).

**FIGURE 7:**
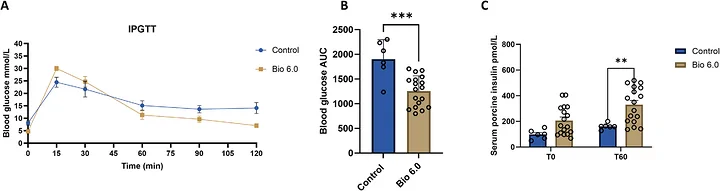
Ten‐week intraperitoneal glucose tolerance test (IPGTT) in diabetic recipients of Bio 6.0 (*n* = 18) or control (*n* = 6) neonatal porcine islet grafts. (A) Blood glucose levels during IPGTT. (B) Area under the curve (AUC) for the blood glucose profiles shown in (A). (C) Serum neonatal porcine islet insulin concentrations measured at fasting (T0) and 60 min after intraperitoneal glucose injection (T60); *** = *p* < 0.001.

At 20 weeks post‐transplantation, mice underwent a second IPGTT to assess long‐term graft function. Bio 6.0 recipients maintained lower blood glucose readings throughout the test compared with controls, although no significant difference in the AUC was observed at this time point (Figure 8A,B). Serum porcine insulin measurements at T60 were higher in Bio 6.0 recipients compared with controls, with a statistically significant increase (*p* < 0.05) (Figure 8D), indicating persistent in vivo insulin secretion from the transplanted Bio 6.0 grafts. Following nephrectomy of the graft‐bearing kidney, total graft insulin content was significantly higher in Bio 6.0 recipients compared with control recipients (*p* < 0.001) (Figure 8C). Removal of the kidney‐bearing grafts resulted in a return to hyperglycemia within one week in all mice, confirming that glycemic control was graft‐dependent. Immunofluorescence staining of the graft‐bearing kidneys confirmed the presence of insulin and glucagon‐positive cells within the grafts of both experimental groups, sections showing abundant insulin‐positive β cell aggregates. Chromogenic immunohistochemistry for insulin further corroborated the insulin content observed in Bio 6.0 grafts relative to controls (Figure 8E).

**FIGURE 8:**
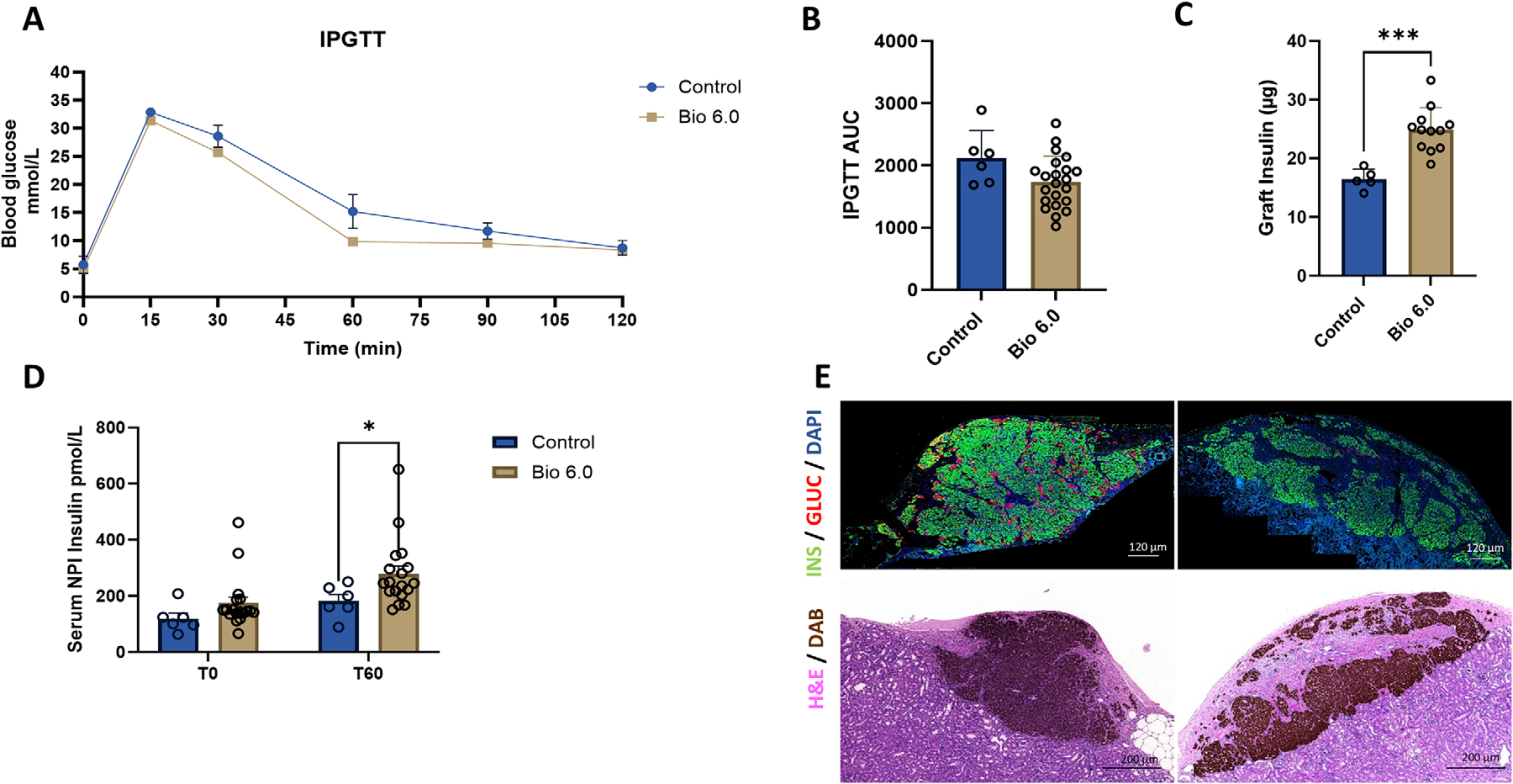
Twenty‐week intraperitoneal glucose tolerance test (IPGTT), graft insulin secretion, and histological characterization of neonatal porcine islet grafts in Bio 6.0 or control recipients. (A) Blood glucose levels during IPGTT in control (*n* = 6) and Bio 6.0 (*n* = 18) groups. (B) Area under the curve (AUC) for the blood glucose profiles shown in (A). (C) Graft‐derived insulin content measured in control (*n* = 5) and Bio 6.0 groups (*n* = 12). (D) Serum neonatal porcine islet insulin concentrations measured at fasting (T0) and 60 min after intraperitoneal glucose injection (T60). (E) Representative immunofluorescence images of graft sections showing co‐localization of insulin and glucagon with nuclear counterstaining by DAPI, and chromogenic immunohistochemistry for insulin using a DAB (3,3′‐diaminobenzidine) substrate; * = *p* < 0.05, *** = *p* < 0.001.

## 4 Discussion

NPIs have the potential to be a promising option for treating T1D using cell replacement therapies [32, 58, 59]. Recent successes in transplanting porcine organs, such as kidneys and hearts, into humans [60, 61, 62], in addition to the recent clinical milestone in which a CRISPR‐engineered porcine kidney successfully supported the renal function in a 60‐year‐old human recipient [33]. This achievement marks the rapidly advancing feasibility of porcine xenotransplantation and strengthens the rationale that NPIs can ultimately deliver a treatment for T1D [32]. Despite this potential, the major roadblock is the absence of a reliable and large‐scale method for NPIs production for clinical use [37, 40, 41, 42]. Overcoming this limitation is essential to unlocking the full therapeutic potential of NPIs transplantation for patients. While vertical wheel bioreactors have proven effective for upscaling growth and directed differentiation of stem cells [46, 47, 49, 63, 64], their application to primary islet tissues has not been fully explored. Here, we show that combining our group's established stepwise enrichment protocol [32, 50, 51] with the PBS vertical wheel bioreactor performs better, especially at high seeding density (6000 NPIs/mL). This density significantly improves functional outcomes compared to both medium‐density bioreactor cultures (4500 NPIs/mL) and classic static suspension systems. This work builds directly upon our previous studies, in which NPIs matured in static suspension cultures showed clear β cell development and functional performance both in vitro and in vivo *[* 50, 52, 57]. However, as with other protocols in the field, substantial loss of β cells and supportive populations during the culture period persisted, ultimately limiting graft yield for clinical scalability [37, 65].

Our previous work laid a solid foundation for the maturation of NPIs in static suspension cultures. Those studies set the bar for successful β cell development and graft functionality, both in vitro and in vivo, setting a methodological benchmark for NPIs as a potential cell replacement therapy for T1D [50, 52, 57]. However, a clear challenge identified across these foundational efforts, one that others in the field have also encountered, is the attrition of cellular material, especially β cells and supportive niche cells, during prolonged culture. This cell loss inevitably reduced the proportion of endocrine cells and limited overall islet yield, posing a hurdle for producing a graft size suitable for clinical transplantation [65].

Building directly on our group's established plate‐based enrichment protocol, the present study introduces high‐density seeding in the PBS Vertical‐Wheel bioreactor as a potential step toward scalable NPIs production. While Corrales et al. and Vanderschelden et al. recommended limiting NPIs culture to 7 days and demonstrated that shortening the protocol to as few as 3 days better preserves islet number and maintains suitability for clinical transplantation [37, 65], the enhanced culture microenvironment provided by the dynamic suspension in the PBS Vertical‐Wheel bioreactor enabled our group's 14‐day enrichment period protocol to yield higher islet recovery, as presented by DNA recovery. The Bio 6.0 condition yielded significantly higher DNA recovery than both the static controls and Bio 4.5 groups, reflecting improved islet survival across the full culture period. The higher DNA recovery was accompanied by a corresponding increase in cellular insulin content, collectively indicating that Bio 6.0 provided a more supportive microenvironment for β cell survival. The higher recovery in Bio 6.0 than in Bio 4.5 may possibly reflect density‐dependent effects on aggregate interactions and local cell–cell communication [66]. At 6000 aggregates/mL, the greater cellular density may have enhanced cell–cell contact and local accumulation of cell signaling molecules and cytokines, thereby supporting NPI survival and limiting cellular attrition during the 14‐day culture period. This is consistent with studies showing that higher cell density promotes pancreatic endocrine differentiation through increased cell–cell contact/aggregation and local signaling [63, 66, 67]. Among the seeding densities tested, 6000 aggregates/mL was the best performing condition.

Focusing on β cell outcomes, our study demonstrates an improvement in β cell content and recovery when compared to earlier studies that utilized static suspension culture from our group as well as other groups, including the well‐established work of Hassouna et al. They reported β cells rising from 20.0% to 41.8% of the islet population after differentiation; however, the absolute recovery of β cells/ pancreas remained unchanged, with differentiated and control groups yielding 8.9 ± 1.8 million and 9.2 ± 2.6 million β cells/pancreas, respectively [50]. Seeberger et al., in their recent comprehensive analysis, found that the percentage of insulin‐positive β cells after differentiation was in the 28%–38% range, depending on the pancreatic lobe, again with no significant increase in absolute β cell yield following in vitro maturation [52]. Lau et al. reported that β cell composition in standard Ham's F‐10 static culture reached only 11.12 ± 1.20% at day 14, and the addition of an optimized islet maturation medium raised this only modestly to 12.20 ± 1.60% [68], suggesting that static culture conditions themselves limit the extent of β cell maturation achievable in vitro. Smith et al. reported that β cell composition was similar in neonatal and juvenile porcine islets, with β cells representing roughly 20% of islet cells in both age groups [69]. These results align well with the β cell yields observed in our control cultures (22.88% ± 2.30), which again confirms the consistency of our differentiation protocol [32, 50, 51].

In this study, we found that while the proportion of β cells remained comparable across all conditions, the actual recovery and β cells/pancreas in the Bio 6.0 group were higher. This means that while the cell population remained constant, better preservation of the β cell compartment through recovering more islets was evident. The increased β cell recovery was accompanied by signs of functional maturation, as evidenced by elevated *PDX1* and insulin gene expression in Bio 6.0 from our PCR analysis. This fits with the role of *PDX1* in marking both β cell lineage commitment and functional advancement [70]. It's also worth noting that the recovery of α cells and CK7‐positive ductal cells remained consistent across all groups, suggesting that the high‐density seeding protocol positively impacts β cell enrichment without skewing overall cell composition.

Metabolic analyses further reinforced this view and aligned well with the previously discussed results. While OCR and ECAR profiles and AUC stayed similar across the groups, the Bio 6.0 group showed a distinctly higher OCR/ECAR ratio, reflecting a more oxidative, mature metabolic phenotype typical of adult β cells. As described by Li et al., mature β cells suppress lactate production and gluconeogenesis through silencing the *LDHA* gene, and their maturation is defined by an increased capacity for glucose‐coupled oxidative phosphorylation, consistent with the elevated OCR/ECAR ratio observed in the Bio 6.0 group [71]. Importantly, this increase in oxidative phosphorylation was not accompanied by higher proton leak, confirming that the mitochondria in these cells remained healthy and functioning efficiently. Taddeo et al. demonstrated that glucose‐stimulated OXPHOS upregulation in functional β cells is specifically ATP‐coupled, whereas increased proton leak is a hallmark of mitochondrial dysfunction [72]. These findings collectively point to genuine metabolic maturation rather than a nonspecific stress response, in line with Walker et al., who stated that sustained coupled OXPHOS is a direct driver of mature β cell identity [73].

Beyond numerical yield and function, we also observed that bioreactor‐derived aggregates were consistently smaller than their static suspension counterparts. This reduction in aggregate size is expected to facilitate more homogeneous diffusion of oxygen and nutrients throughout each aggregate, supporting more uniform enrichment and survival. Honarpisheh et al. demonstrated that controlled re‐aggregation of NPIs into smaller clusters improved endocrine cell organization and vascular density after transplantation, leading to better graft integration [74]. In agreement with these observations, Rout et al. systematically concluded that small islets with diameters below 125 µm showed superior post‐transplant outcomes in terms of higher viability, greater glucose‐stimulated insulin secretion, and improved engraftment. Whereas larger islets are prone to central hypoxia, necrosis, and functional decline following isolation [54, 75]. Taken together, these observations indicate that the smaller, more uniform aggregates generated in our bioreactor cultures are compatible with islet size ranges associated with improved graft survival and function in transplantation studies.

The specific culture microenvironment exerted a clear influence on primary NPIs survival and gene expression. In a static culture condition, islets have been reported to adhere to the vessel surface, driving epithelial‐to‐mesenchymal transition (EMT) and dedifferentiation, a process marked by elevated levels of the ductal/progenitor marker *SOX9* [39, 40, 76]. The dynamic suspension culture in the bioreactor eliminated this adherence to the vessel surface, preserving 3D aggregate integrity and significantly downregulating *SOX9*, which prevents progression to an uncommitted ductal/progenitor phenotype [77]. On the other hand, *NKX6.1* expression, an essential master transcription factor for mature β‐cell functional identity [78] was maintained in Bio 6.0 at control levels, unlike the decrease observed in the Bio 4.5 condition. This was associated with a marked upregulation in *INS*, *GLUC*, and *PDX1* in the Bio 6.0 group. The observed downregulation of PAX6 in both bioreactor groups may reflect cell‐state changes and transcriptional tuning induced by the dynamic culture conditions [78]. While individual transcript markers provide limited observation of active ß cell regulatory state, the preservation of *NKX6.1* and strong upregulation of *PDX1*, combined with superior in vivo glycemic control and metabolic function, demonstrate functional NPIs maturation in dynamic culture conditions.

These findings are strongly supported by recent studies utilizing vertical wheel bioreactors and similar dynamic 3D suspension systems. For instance. Dadheech et al. demonstrated that scale‐up production of human iPSC‐derived islets in Vertical Wheel bioreactors enhanced β cell composition, glucose‐responsive insulin release, and reversed diabetes in STZ‐treated mice, with single‐cell RNA sequencing confirming transcriptional maturity comparable to adult islets [49, 64]. Similar reports show that dynamic, three‐dimensional culture not only protects against EMT and dedifferentiation observed in static or 2D conditions, but actually accelerates the acquisition of functional β cell identity [79, 80, 81, 82, 83]. Collectively, these comparisons indicate that the gene expression and maturation advantages observed in our Bio 6.0 group align closely with the results recently published using bioreactor approaches [48, 49, 64], and that dynamic, vertical wheel systems stand out as a preferred strategy for directing efficient, potentially scalable, and functional NPIs maturation in vitro.

Building on the in vitro analysis, our findings were further reinforced and extended when aggregates from the Bio 6.0 group were transplanted into immunodeficient diabetic mice, which continued to show improved function. Following transplantation under the kidney capsule, the bioreactor group demonstrated accelerated glycemic recovery and greater graft cellular insulin content when compared to the control group. This improved functional performance of the bioreactor group was further validated by IPGTT results at 10 weeks post‐transplantation. Mice transplanted from the bioreactor group showed better tolerance to intraperitoneal glucose injections when compared to the control group. The bioreactor group also showed higher NPIs serum insulin after one hour following the glucose injection at T60 when compared to the control group, again bringing back the finding of more β cell recovery, β cell/pancreas, and total insulin content in the smaller bioreactor aggregates before transplantation when compared to control. This elevated glucose‐stimulated serum insulin at T60 is also consistent with the enhanced metabolic activity observed in vitro, where the Bio 6.0 group demonstrated a superior OCR/ECAR ratio indicative of a more responsive and functional β cell phenotype. These in vivo data thus provide functional validation of the in vitro observations and are in line with findings from Cuesta‐Gomez et al., who demonstrated that glucose‐responsive porcine insulin secretion is observed at both 8 and 20 weeks post‐transplant, with serum insulin levels at T60 serving as a reliable indicator of graft maturity and functional advancement [55].

As the control group transplants matured over time, both groups showed comparable areas under the curve in IPGTT profiles and a smaller difference between T0 and T60 at 20 weeks post‐transplantation, indicating that both groups ultimately reached a state of metabolic homeostasis. This convergence over time aligns with the well‐established trajectory of NPIs maturation in vivo, previously reported by our group and other groups as well [50, 52, 55, 57, 84, 85]. Importantly, even as overall glycemic control converged between groups, the total insulin content extracted from the grafts at 20 weeks post‐transplantation remained higher in the bioreactor group. This finding could reflect a greater and more mature β cell mass within the explanted grafts, which may have led to superior graft function. This mirrors the seminal work of Korbutt et al. (1996), which first demonstrated a 20‐ to 30‐fold increase in cellular insulin content in NPIs grafts by 14 weeks post‐transplantation under the kidney capsule of diabetic nude mice [32].

The in vivo environment offers a complex confluence of physiological signals unavailable in vitro, including glucose‐responsive paracrine cues, endocrine hormones, and direct revascularization [86]. Upon transplantation into diabetic recipients, the hyperglycemic environment itself becomes a potent stimulus for NPIs maturation and β cell expansion. Trivedi et al. clearly demonstrated that β cell proliferation is significantly higher in grafts of diabetic recipients with persistent hyperglycemia compared to normoglycemic recipients, a stimulatory influence of elevated glucose levels that drives both β cell proliferation and active neogenesis [87]. Similarly,  Omer et al. showed that encapsulated NPIs underwent substantial in vivo maturation over time, with a 10‐fold increase in insulin content and a 3.2‐fold increase in the ratio of β cell area to total cellular area at 20 weeks post‐transplantation compared to pre‐transplant NPIs, confirming that NPIs retain significant post‐transplant enrichment capacity [88]. These reported findings also came in line with Chun Li et al., who stated that the diabetic in vivo environment could be a potential trigger for β cell progenitor propagation and differentiation [89]. The robust β cell immunofluorescence observed in both bioreactor and control explanted grafts highlights the remarkable plasticity of NPIs and raises compelling questions about the relative power of the in vivo diabetic niche to allow for the expansion and maturation of the β cell population in the absence of autoimmunity. The superior total graft insulin content at 20 weeks in the bioreactor group suggests that the in vitro head start provided by the PBS Vertical Wheel Bioreactor, higher insulin recovery, higher β cell recovery, β cell/pancreas, improved metabolic maturity, and smaller aggregate size meaningfully potentiate the response to the in vivo niche, ultimately yielding an enhanced functional graft. Collectively, these findings highlight that the in vivo niche is a potent and indispensable driver of NPIs maturation that acts in concert with, and is enhanced by, superior in vitro priming.

This NPI high‐density culture technique integrating the PBS Vertical‐Wheel Bioreactor provides an important step toward assessing the potential future scalability and clinical translation of this approach. Future studies should expand this work to larger‐scale bioreactor systems and include systematic media‐volume and nutrient‐demand calculations, assessment of batch‐to‐batch consistency, and detailed evaluation of overall process yield. Ultimately, validation in large preclinical animal model will be needed to determine whether bioreactor‐enriched NPIs can support durable graft survival, revascularization, and long‐term glycemic control. This stepwise strategy, preserving an effective enrichment backbone while progressively increasing the potential for scalability, reproducibility, and translational performance, may help advance the platform toward clinical application.

## Conflicts of Interest

The authors of this manuscript have no conflicts of interest. All authors acknowledge participation in the Transplant Peer Review Network and complied with the journal's author guidelines and policies.

## Generative AI Statement

AI‐assisted tools were used to enhance language and clarity during the drafting and editing of the manuscript; all content was reviewed and verified by the authors, who take full responsibility for its accuracy and integrity.
